# Multifunctional liposome for photoacoustic/ultrasound imaging-guided chemo/photothermal retinoblastoma therapy

**DOI:** 10.1080/10717544.2022.2032876

**Published:** 2022-02-14

**Authors:** Meng Li, Xintong Bian, Xu Chen, Ningke Fan, Hongmi Zou, Yixi Bao, Yu Zhou

**Affiliations:** a Department of Clinical Laboratory, The Second Affiliated Hospital of Chongqing Medical University, Chongqing, PR China;; b Key Laboratory of Clinical Laboratory Diagnostics (Ministry of Education), College of Laboratory Medicine, Chongqing Medical University, Chongqing, PR China;; c Zhongshan Ophthalmic Center, State Key Laboratory of Ophthalmology, Sun Yat-sen University, Guangzhou, PR China;; d Department of Ophthalmology, The Second Affiliated Hospital of Chongqing Medical University, Chongqing, PR China

**Keywords:** Nanoplatform, dual-modal imaging, doxorubicin, photothermal therapy, retinoblastoma

## Abstract

Retinoblastoma (RB) is a malignant intraocular neoplasm that occurs in children. Diagnosis and therapy are frequently delayed, often leading to metastasis, which necessitates effective imaging and treatment. In recent years, the use of nanoplatforms allowing both imaging and targeted treatment has attracted much attention. Herein, we report a novel nanoplatform folate-receptor (FR) targeted laser-activatable liposome termed FA-DOX-ICG-PFP@Lip, which is loaded with doxorubicin (DOX)/indocyanine green (ICG) and liquid perfluoropentane (PFP) for photoacoustic/ultrasound (PA/US) dual-modal imaging-guided chemo/photothermal RB therapy. The dual-modal imaging capability, photothermal conversion under laser irradiation, biocompatibility, and antitumor ability of these liposomes were appraised. The multifunctional liposome showed a good tumor targeting ability and was efficacious as a dual-modality contrast agent both *in vivo* and *in vitro*. When laser-irradiated, the liposome converted light energy to heat. This action caused immediate destruction of tumor cells, while simultaneously initiating PFP phase transformation to release DOX, resulting in both photothermal and chemotherapeutic antitumor effects. Notably, the FA-DOX-ICG-PFP@Lip showed good biocompatibility and no systemic toxicity was observed after laser irradiation in RB tumor-bearing mice. Hence, the FA-DOX-ICG-PFP@Lip shows great promise for dual-modal imaging-guided chemo/photothermal therapy, and may have significant value for diagnosing and treating RB.

## 1. Introduction

Retinoblastoma (RB) is a relatively common malignant neoplasm of the eye seen in young children (Pandey, 2014), with an incidence rate of about 1:15,000 to 1:20,000 (Kivelä, 2009). In lower-income countries, only 40% of RB patients survive (Canturk et al., 2010), compared to 95% in developed countries (Broaddus et al., 2009; MacCarthy et al., 2009). The reasons for this discrepancy are related to delays in diagnosis and treatment rather than intrinsic differences in tumor types (Chawla et al., 2016). Therefore, there is an urgent need to develop imaging methods that facilitate early diagnosis as well as new and effective treatments. Nanomaterials, including liposomes and organic, inorganic, and metal nanoparticles, have been applied to optical (Lesiak et al., 2019), photoacoustic (PA) (Guo et al., 2019), ultrasound (US) (Wang et al., 2018), magnetic resonance (MR), and radionuclide (Ahmad et al., 2019) tumor imaging and targeting drug delivery. In addition, drug release can be used in conjunction with photodynamic (Zhu et al., 2018) or photothermal (Wang et al., 2018) therapy. Thus, the utilization of nanoparticle technology provides a new and effective means of coordinating imaging and therapy for RB.

In the past few decades, advances in nanotechnology have opened new opportunities for the treatment of many major diseases. Liposomes are frequently used for the delivery of antitumor nanomedicine, and this technology has proved highly successful not only for delivery but also in terms of biosafety and biodegradability (Chen et al., 2017). Liposome technology has been approved by the US Food and Drug Administration (FDA) and is widely used in the pharmaceutical industry. Another FDA-approved compound, PEG, functions as a biocompatible polymer, and when mixed with ordinary liposomes that can reduce the intake of reticuloendothelial system, prolonging the circulatory half-lives of the liposomes and nanocomplexes.

Indocyanine green (ICG) is a fluorescent dye created by Kodak Research Laboratories in 1955 and approved by the FDA in 1956 for human imaging and medical applications (Feindel et al., 1967). ICG is frequently used in ophthalmology to evaluate retinal disorders and tumors. The dye absorbs in the near-infrared (NIR) part of the spectrum at approximately 800 nm, and is thus subject to good photothermal conversion (Kuo et al., 2012). Due to this, it has wide applications in photothermal therapy (PTT) and has been applied as a contrast agent in PA imaging (Hu et al., 2016).

Folic acid is a water-soluble B vitamin and micronutrient (Matsuzaki et al., 2008) that binds to specific folic acid receptors (FRs) (Chen et al., 2013). Expression of FRs is markedly upregulated in tumor tissues (Toffoli et al., 1997) and they have also been shown to be overexpressed in RB (Kansara et al., 2008). Nanoparticles coated with folic acid can target FR-overexpressing cells (Assaraf et al., 2014) and through the binding of folic acid to FRs, can be taken up by the cells.

Perfluoropentane (PFP) is a biocompatible perfluorocarbon compound. It boils at approximately 29 °C under one atmosphere, and forms microbubbles on exposure to heat or US (Zhao et al., 2021). Due to these properties, the application of PFP in nanoparticle formation has become popular (Jinsui et al., 2018; Xie et al., 2018; Wu et al., 2019; Zhang et al., 2019; Li et al., 2021). When encapsulated by nanoparticles, liquid PFP vaporizes to microbubbles by optical droplet vaporization (ODV) (Zhang et al., 2019), which enhances intensity in US imaging (Cheng et al., 2013). The microbubbles, in turn, augment cell permeability and enhance drug release, thus reducing the side effects of the drugs (Yang et al., 2014). Tumor cells may also be rendered unstable by the PFP-induced cavitation effect under laser irradiation, indicating an additional application to enhance the efficacy of antitumor therapies in conjunction with PTT or chemotherapy.

**Scheme 1.**
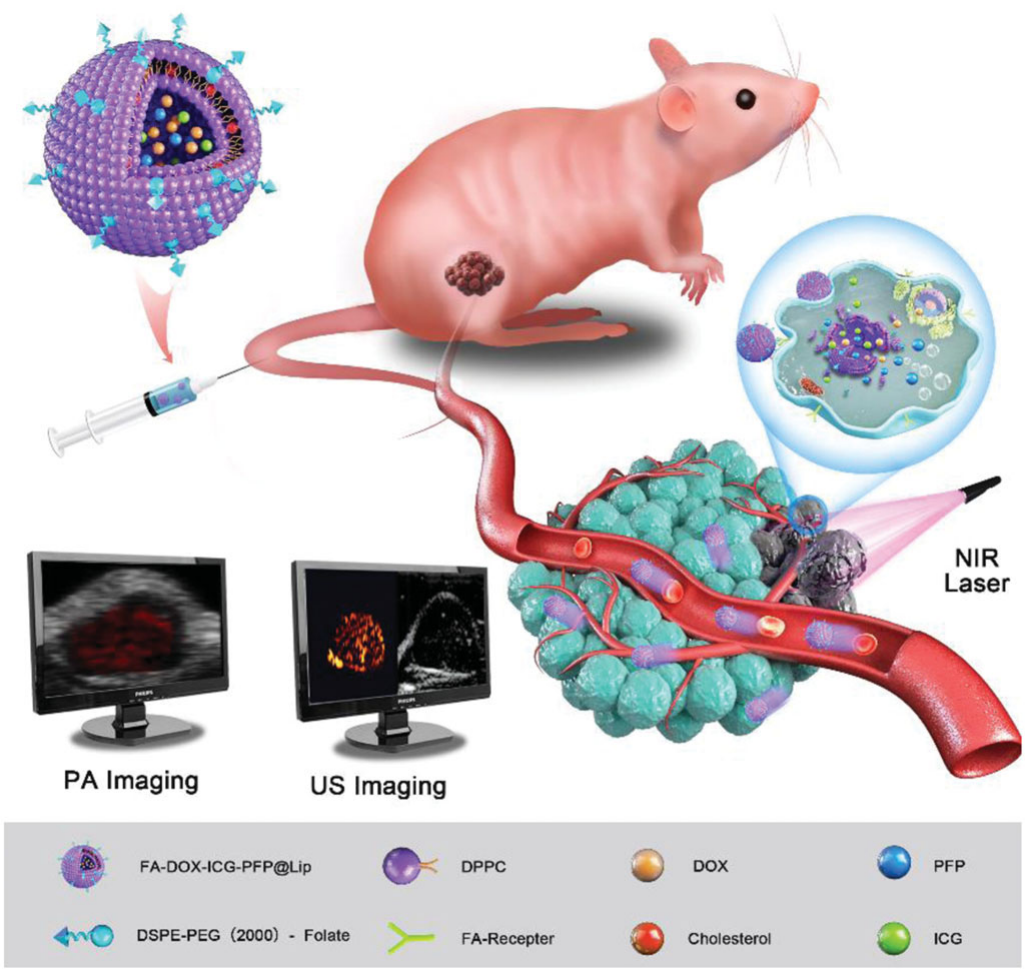
Schematic illustration of FA-DOX-ICG-PFP@Lip and corresponding dual-modal imaging for the guidance of synergistic photothermal and chemical therapy for Y79 retinoblastoma.

Here, FA-DOX-ICG-PFP@Lip nanoparticles were fabricated and their ability to target FR-expressing RB cells was investigated. As these nanoparticles have extended circulatory half-lives and are able to evade macrophage removal, they can accumulate in tumor cells with few side effects. Using 808 nm laser irradiation, the ICG within the liposomes can convert light energy to heat, causing both severe damage to the cell and ODV of PFP leading to the release of doxorubicin (DOX). In addition to these antitumor effects, we have found that these liposomes, due to both the PA imaging properties of ICG and the phase-shifting capacity of PFP, could be used effectively as dual-modality contrast agents for both PA and US imaging. This liposome system thus augments cellular uptake and antitumor action for drug delivery using a combination of laser activation and PTT (Scheme 1).

## 2. Materials and methods

### 2.1. Materials

1,2-Distearoyl-sn-glyc-ero-3-phosphoethanolamine-N-[folate(polyethyleneglycol)-2000] (DSPE-PEG (2000)-folate), cholesterol, PFP, ICG, DOX, 1,1′-dioctadecyl-3,3,3′,3′-tetramethylindotricarbocyanine iodide (DiR), Hoechst 33342, were brought from Sigma-Aldrich Co. (St. Louis, MO). 1,2-Dihexadecanoyl-rac-glycero-3-phoshocoline (DPPC) was purchased from Xi’an Ruixi Biological Technology (Xi’an, China). Chloroform (CHCl_3_) was purchased from Chongqing East Chemical Industry Ltd., Co. (Chongqing, China). Molecular biology-grade agarose was purchased from Thermo Fisher Scientific (Waltham, MA). Monoclonal antibodies against folic acid in mice (primary antibody) were obtained from Beijing Hapten and Protein Biomedical Institute (Beijing, China). FITC-labeled anti-mouse IgG antibodies in sheep (second antibody) were purchased from Abcam (Waltham, MA). The human RB cell line Y79 and human umbilical vein endothelial cells (HUVECs) were purchased from the China Center for Type Culture Collection (Wuhan, China). The cell counting kit-8 (CCK-8) assay was obtained from Dojindo Laboratories (Kumamoto, Japan). Deionized water obtained from the Millipore system (Direct-Q5, FRA, Billerica, MA) was used in all preparations.

### 2.2. Preparation of FA-DOX-ICG-PFP@Lip

For the targeted FA-DOX-ICG-PFP@Lip, DPPC, DSPE-PEG (2000)-folate, and cholesterol were dissolved in CHCl_3_ at a weight ratio of 5:2:3. The mixture was treated with a rotary vacuum evaporator for 2 h to evaporate CHCl_3_ and to form a thin lipid film, followed by hydration of the film with phosphate-buffered saline (PBS, pH 7.4). Meanwhile, 100 µL DOX (10 mg/mL), 100 µL ICG (10 mg/mL), and 200 µL PFP were emulsified using a sonicator (VCX130; Sonics and Materials, Newton, CT) with a power of 100 W for 2 min (5 s on, 5 s off). This DOX-ICG-PFP mixture was then dropped onto the lipid film. The suspension was re-emulsified by sonication with a power of 125 W for 6 min (5 s on, 5 s off), centrifuged (8000 rpm, 5 min), and washed with water. The centrifugation and washing steps were repeated three times. The resultant FA-DOX-ICG-PFP@Lip was stored at 4 °C. The preparation of DOX-ICG-PFP@Lip was the same, except that DSPE-PEG (2000)-folate was substituted with DSPE-PEG (2000). When preparing fluorescence-labeled liposomes, the fluorescent dye DiR (1 mg) was included in the organic solution, otherwise, the steps were the same.

### 2.3. Characterization of FA-DOX-ICG-PFP@Lip

The morphology of FA-DOX-ICG-PFP@Lip was observed by transmission electron microscopy (TEM) (Hitachi, Tokyo, Japan). The size distribution and surface charge were measured with a Laser Particle Size Analyzer System (Zetasizer Nano ZS90, Malvern Instruments, Malvern, UK). The encapsulation efficiency (EE) and loading capacity (LC) of DOX and ICG were measured by ultraviolet spectrophotometry (Lambda 950; PerkinElmer, Waltham, MA), and calculated according to the following formulae:

$$\text{EE}\;(\text{DOX})=\frac{t\text{otal}\;\text{mass}\;\text{of}\;\text{added}\;\text{DOX}-\text{mass}\;\text{of}\;\text{DOX}\;\text{in}\;\text{supernatant}}{\text{total}\;\text{mass}\;\text{of}\;\text{added}\;\text{DOX}}\times 100\%$$

$$\text{LC}\;(\text{DOX})=\frac{\text{total}\;\text{mass}\;\text{of}\;\text{added}\;\text{DOX}-\text{mass}\;\text{of}\;\text{DOX}\;\text{in}\;\text{supernatant}}{m\text{ass}\;\text{of}\;\text{liposome}}\times 100\%$$

$$\text{EE}\;(\text{ICG})=\frac{t\text{otal}\;\text{mass}\;\text{of}\;\text{added}\;\text{ICG}-m\text{ass}\;\text{of}\;\text{ICG}\;\text{in}\;\text{supernatant}}{t\text{otal}\;\text{mass}\;\text{of}\;\text{added}\;\text{ICG}}\times 100\%$$

$$\text{LC}\;(\text{ICG})=\frac{t\text{otal}\;\text{mass}\;\text{of}\;\text{added}\;\text{ICG}-\text{mass}\;\text{of}\;\text{ICG}\;\text{in}\;\text{supernatant}}{\text{mass}\;\text{of}\;\text{liposome}}\times 100\%$$

### 2.4. Assessment of folate on the shell of FA-DOX-ICG-PFP@Lip

Binding of folate on the surface of the FA-DOX-ICG-PFP@Lip was examined by immunofluorescence. The FA-DOX-ICG-PFP@Lip was first blocked with BSA to prevent nonspecific anti-folate antibody binding. The FA-DOX-ICG-PFP@Lip suspension was then incubated with the primary antibody at a ratio of 500:1 (v:v) for 4 h at room temperature. After washing in PBS, the liposomes were centrifuged (8000 rpm, 5 min) and incubated with the secondary antibody at a ratio of 100:1 (v:v) for 1 h at room temperature, followed by further washing and separation. Then, liposomes were washed and separated using the same steps as for the primary antibody. Finally, the binding ability of folate was examined by confocal laser-scanning microscopy (CLSM; A1R-Si; Nikon, Tokyo, Japan) and the folate loading rate was measured using flow cytometry.

### 2.5. The effects on temperature and phase change induced by NIR laser irradiation

To evaluate the capacity of FA-DOX-ICG-PFP@Lip for PTT and phase transition, saline solutions containing different concentrations of FA-DOX-ICG-PFP@Lip (0.625, 1.25, 2.5, and 5.0 mg/mL) were exposed to NIR laser irradiation (808 nm) at 1 W/cm^2^ for 5 min. Changes in temperature were measured with a thermal imaging camera (Fotric 226; ZXF Laboratories, Allen, TX) and phase transitions of FA-DOX-ICG-PFP@Lip were visualized under an inverted microscope (Olympus BX51, Olympus Corp., Tokyo, Japan).

### 2.6. *In vitro* laser-triggered DOX release

Drug release from FA-DOX-ICG-PFP@Lip at different times with and without NIR laser irradiation was investigated. Two identical samples of FA-DOX-ICG-PFP@Lip were placed in dialysis bags which were then placed in deionized water and shaken at 100 rpm. One-milliliter aliquots were removed at specified times and replaced with 1 mL deionized water. In the FA-DOX-ICG-PFP@Lip + laser group, the material in the bag was irradiated by NIR laser (808 nm, 1 W/cm^2^) for 5 min at 1 h after the aliquot removal, after which the material was treated the same as the controls. The released DOX in the two groups was quantified by high-performance liquid chromatography (HPLC; LC-2010A HT; Shimadzu, Kyoto, Japan).

### 2.7. *In vitro* dual-mode imaging performance of FA-DOX-ICG-PFP@Lip

The potential of FA-DOX-ICG-PFP@Lip as contrast agents for PA and US imaging was assessed using an agar gel model. The PA average values of the six groups (0.625, 1.25, 2.5, and 5 mg/mL FA-DOX-ICG-PFP@Lip; 5 mg/mL DOX-ICG-PFP@Lip and saline) were detected and analyzed by a PA imaging system (Vevo Lazr; Visual Sonics, Toronto, Canada). B-mode and contrast-enhanced US (CEUS) images of the six groups were acquired by ultrasonography (MyLab 90; Esaote, Genoa, Italy) and the average echo intensity was determined with US imaging-analysis software (DFY; Institution of US Imaging of Chongqing Medical University, Chongqing, China) after 5 min of irradiation with 1 W/cm^2^ 808 nm laser.

### 2.8. Cell culture and establishment of tumor model

The human RB cell line Y79 and HUVECs were cultured in RPMI-1640 medium supplemented with 10% fetal bovine serum (FBS) and 1% penicillin–streptomycin in a humidified incubator at 37 °C under 5% CO_2_. Cells in the logarithmic growth phase were used for the experiments. Female BALB/c nude mice (6–8 weeks old, weighing 18–20 g) were obtained from and maintained in the Laboratory Animal Center of Chongqing Medical University. All experiments and procedures were carried out under guidelines approved by the Institutional Animal Care and Use Committee of Chongqing Medical University. To establish the xenograft model, Y79 cells in PBS (1 × 10^6^ cells per mouse) were injected subcutaneously into the flanks of the mice.

### 2.9. Measurement of cytotoxicity *in vitro*

Y79 and HUVECs (5 × 10^3^ cells per well) were plated in 96-well plates and cultured for 24 h. The media were then replaced with fresh medium containing FA-ICG-PFP@Lip (DOX-free) at different concentrations (0.625, 1.25, 2.5, and 5 mg/mL). The cells were allowed to grow for 6, 12, or 24 h, after which 20 μL of CCK-8 reagent were added to each well and incubated for 1 h. Absorbances at 450 nm were read in a microplate reader (ELX800; BioTek Instruments, Winooski, VT) and the cell viability was calculated as

$$\frac{A_{450}\;\text{of}\;\text{treated}\;\text{cells}}{A_{450}\;\text{of}\;\text{control}\;\text{cells}}\times 100\%$$

### 2.10. Cellular uptake

Y79 cells were plated in six-well plates (2 × 10^5^ cells per well) and grown in medium with or without folic acid. The same doses of FA-DOX-ICG-PFP@Lip or DOX-ICG-PFP@Lip were then added to specific wells and incubated for 3 h. The nuclei were stained with Hoechst 33342 for 15 min after which the cells were washed three times with PBS and observed under CLSM (A1R-Si; Nikon, Tokyo, Japan).

### 2.11. Anticancer effect *in vitro*

Y79 cells were plated in 96-well plates (5 × 10^3^ cells per well) and allowed to grow for 24 h under different treatments: control, laser only, DOX only, FA-DOX-ICG-PFP@Lip, FA-ICG-PFP@Lip + laser, DOX-ICG-PFP@Lip + laser, and FA-DOX-ICG-PFP@Lip + laser. The indicated groups were treated with NIR laser irradiation (808 nm) at 1 W/cm^2^ for 5 min and the cell viability was assessed using the CCK-8 assay.

### 2.12. Assessment of biocompatibility

To evaluate the biosafety of FA-DOX-ICG-PFP@Lip, 20 female nude mice were intravenously injected with 200 μL of different treatments (saline only, 2.5 mg/mL FA-DOX-ICG-PFP@Lip). The mice were killed on days 1, 7, or 15 after the injection, and blood samples were taken for routine blood and serum biochemistry assessments.

### 2.13. *In vivo* biodistribution of FA-DOX-ICG-PFP@Lip

Live fluorescence imaging was used to assess the targeting of FA-DOX-ICG-PFP@Lip *in vivo*. Ten Y79 tumor-bearing mice were assigned to two groups (*n* = 5) and injected with DiR-labeled FA-DOX-ICG-PFP@Lip or DOX-ICG-PFP@Lip (200 μL, 2.5 mg/mL) through the tail vein. The mice were then imaged using a fluorescence system (Fx7 IR Spectra; Vilber Lourmat, Collégien, France) at specified times. On sacrifice, the tumors and principal organs (heart, liver, spleen, lungs, and kidneys) were evaluated by fluorescence imaging.

### 2.14. *In vivo* dual-mode imaging capability

The Y79 tumor-bearing mice were randomly divided into two groups (FA-DOX-ICG-PFP@Lip and DOX-ICG-PFP@Lip) (*n* = 5). The liposomes (200 μL, 2.5 mg/mL) were injected intravenously, and the PA images and values at varying times (0, 1, 3, 6, 12, and 24 h) were recorded. US images of the tumors were also obtained before and after 808 nm laser irradiation, with DFy used to analyze echo intensity.

### 2.15. *In vivo* anticancer evaluation

Y79 tumor-bearing mice were randomly assigned to seven groups (control, laser only, DOX only, FA-DOX-ICG-PFP@Lip, FA-ICG-PFP@Lip + laser, DOX-ICG-PFP@Lip + laser, FA-DOX-ICG-PFP@Lip + laser) (*n* = 5). The mice received intravenous injections of saline (200 μL), free DOX (at the liposome-loaded dose), DOX-ICG-PFP@Lip, FA-ICG-PFP@Lip, or FA-DOX-ICG-PFP@Lip. After 3 h, the tumors were irradiated using the 808 nm laser for 5 min at 1 W/cm^2^. The temperature of the tumor area was determined using a thermal imaging camera during the irradiation. The body weights and tumor volumes of the mice were assessed every two days, and the tumor volumes were determined as *V*=π/6×*L*×*W*×*D*. At the completion of the experiment, the mice were sacrificed and the tumors were excised and fixed (4% polyoxymethylene) for histological analysis including hematoxylin–eosin (H&E) staining, TdT-mediated dUTP nick-end labeling (TUNEL), and proliferating cell nuclear antigen (PCNA) staining. The principal organs (heart, liver, spleen, lung, and kidney) were removed, fixed, and stained with H&E.

### 2.16. Statistical analysis

All data were presented as means ± SD. ANOVA and *t*-tests were used to analyze the statistical significance of the experimental data by using SPSS 23.0 software (IBM Corp., Armonk, NY). Significance was defined as *p<.05, **p<.01.

## 3. Results and discussion

FA-DOX-ICG-PFP@Lip is a unique folic acid-modified laser-responsive nanoliposome constructed with chemotherapeutic drug/photosensitizer/phase-change material. It has potential applications both for dual-modal imaging and, in combination with chemo or PTT, for tumor ablation. The folic acid-containing liposomes bind to FRs on RB cells and accumulate due to the enhanced permeability and retention (EPR) effect (Sun et al., 2014; Wang et al., 2016; Kalyane et al., 2019). When the cells are laser-irradiated at 808 nm, the ICG in the liposomes converts the energy to heat which may in itself destroy the tumor cell and also trigger a phase change in PFP, leading to DOX release. Microbubbles produced by ICG and PFP allow the use of photoacoustic/ultrasound (PA/US) dual-modal imaging for the guidance of antitumor chemotherapy or PTT (Scheme 1).

### 3.1. Preparation and characterization of FA-DOX-ICG-PFP@Lip

**Figure 1.**
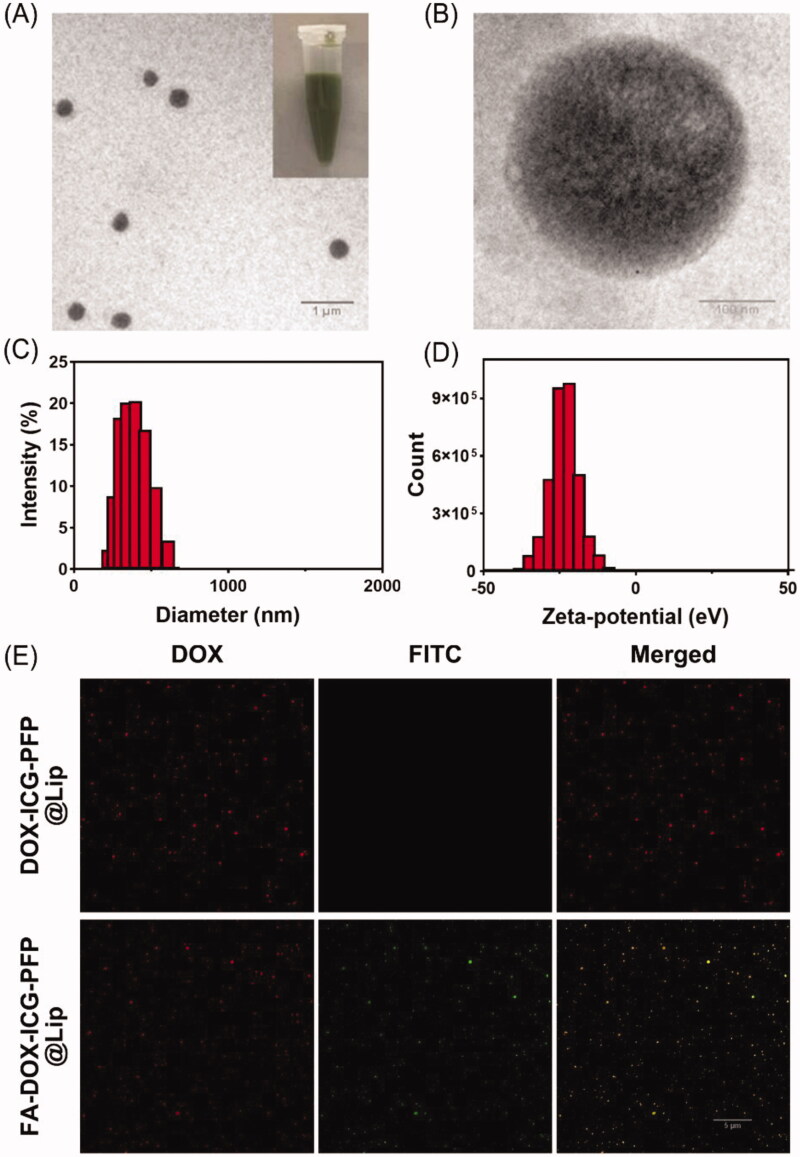
Morphology and characterization of liposomes. (A) Transmission-electron micrograph of FA-DOX-ICG-PFP@Lip. (Inset) Images of the FA-DOX-ICG-PFP@Lip suspension. (B) High-magnification transmission-electron micrograph of FA-DOX-ICG-PFP@Lip. (C) Size distribution of FA-DOX-ICG-PFP@Lip. (D) Zeta potentials of FA-DOX-ICG-PFP@Lip. (E) The detection of the folate on the surface of FA-DOX-ICG-PFP@Lip by laser scanning confocal microscopy.

FA-DOX-ICG-PFP@Lip was produced using a double-emulsion process and US emulsification. The synthesized structure is described in Scheme 1. The morphology of the FA-DOX-ICG-PFP@Lip was examined by TEM. As seen in Figure 1(A,B), the liposome showed a spherical shape and homogenous size. The mean diameter of the FA-DOX-ICG-PFP@Lip was determined by dynamic light scattering and was found to be 309.4 ± 16.7 nm (Figure 1(C)), which matched well with the TEM findings. This size guarantees that the liposomes will be able to penetrate the gaps between endothelial cells in the tumor vasculature. The mean zeta potential of FA-DOX-ICG-PFP@Lip was –20.9 ± 8.6 mV (Figure 1(D)). The negatively charged surface can prolong the blood circulation of FA-DOX-ICG-PFP@Lip and reduce the proton-sponge effect (Nel et al., 2009; Chen et al., 2017). The EE and LC of DOX and ICG were also determined (Table 1).

**Table 1.** Encapsulation efficiencies (EEs) and loading capacities (LCs) of DOX and ICG.

Reagent	EE (%)	LC (%)
DOX	62.04 ± 5.10	6.20 ± 0.51
ICG	91.85 ± 2.98	9.19 ± 0.29

The ability of the folate on the liposome to bind to the FRs on the cell surface is essential for targeting the liposomes to the tumor. We thus investigated whether this function was damaged during the liposome assembly, using immunofluorescence. The DOX-containing liposome appears red under CLSM, while the FA-DOX-ICG-PFP@Lip is seen as green due to the FITC conjugated with the secondary antibody against the anti-folate primary antibody. CLSM analysis showed retention of the folate–FR interaction (Figure 1(E)), indicating that FA-DOX-ICG-PFP@Lip was able to target the FRs.

### 3.2. *In vitro* photothermal effect and phase-changing ability

**Figure 2.**
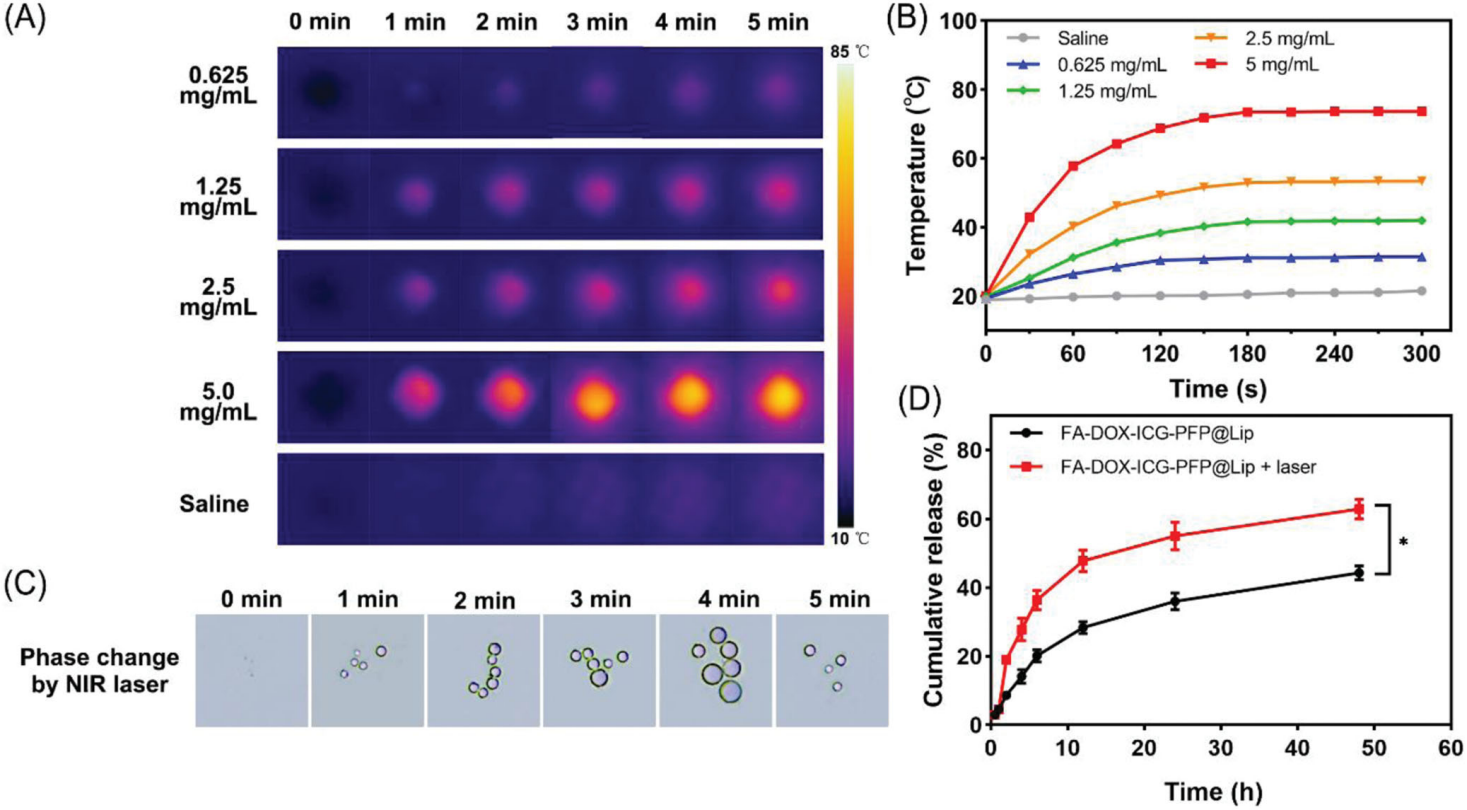
Photothermal effects, phase changes, and drug-release behavior of FA-DOX-ICG-PFP@Lip. (A) Infrared thermal images of FA-DOX-ICG-PFP@Lip at different concentrations irradiated by laser at 1 W/cm^2^ for 5 min. (B) Temperature–time curves of FA-DOX-ICG-PFP@Lip at different concentrations irradiated by laser at 1 W/cm^2^ for 5 min. (C) Micrographs showing phase transition of FA-DOX-ICG-PFP@Lip after exposure to 808 nm NIR laser irradiation at different times. (D) Release of DOX from FA-DOX-ICG-PFP@Lip in the presence or absence of laser irradiation (*p<.05).

To evaluate the photothermal capabilities of FA-DOX-ICG-PFP@Lip, we monitored temperature changes during laser irradiation at 808 nm with a thermal imaging camera. The infrared thermal images and real-time temperature curves (Figure 2(A,B)) showed temperature increases in correspondence with concentration (0.625, 1.25, 2.5, and 5 mg/mL), while no temperature change occurred in the saline. As seen in the figure, the temperature rapidly exceeded 42 °C during the short irradiation time in the 2.5 and 5 mg/mL groups; this is a crucial temperature point for triggering cell damage (Cherukuri et al., 2010). In addition, cancer cells are more susceptible to heat damage than normal cells due to poor heat dissipation resulting from vascular deficiencies (Redolfi Riva et al., 2014), indicating that FA-DOX-ICG-PFP@Lip has potential for tumor ablation.

As changes in phase occur in response to temperature elevation, the transition from liquid to gas was evaluated for FA-DOX-ICG-PFP@Lip. Phase changes in FA-DOX-ICG-PFP@Lip during irradiation (808 nm, 1.0 W/cm^2^, 5 min) were monitored by microscopy. As shown in Figure 2(C), while no microbubbles were seen initially, the numbers of microbubbles increased significantly in response to the irradiation, disappearing gradually when the irradiation was halted. These observations indicate that FA-DOX-ICG-PFP@Lip has satisfactory microbubble-producing capabilities under these conditions and would function well as contrast agents in US imaging.

### 3.3. Laser-triggered drug release

The satisfactory efficiencies observed for photothermal conversion and phase change in the liposomes suggested that laser irradiation could elicit drug release. As shown in Figure 2(D), DOX release did not differ between the experimental and control groups before irradiation. However, after laser irradiation, a sharp increase in release was observed in the FA-DOX-ICG-PFP@Lip + laser group, while the cumulative release ratio increased slowly and constantly in the FA-DOX-ICG-PFP@Lip group. These findings indicate that DOX is effectively released from the FA-DOX-ICG-PFP@Lip in response to radiation. This would lead to an elevated drug concentration at the tumor site, resulting in improved therapeutic effects with fewer adverse effects (He et al., 2017). These findings suggest the potential for using FA-DOX-ICG-PFP@Lip as drug delivery vehicles for precise RB therapy.

### 3.4. *In vitro* dual-modal imaging

**Figure 3.**
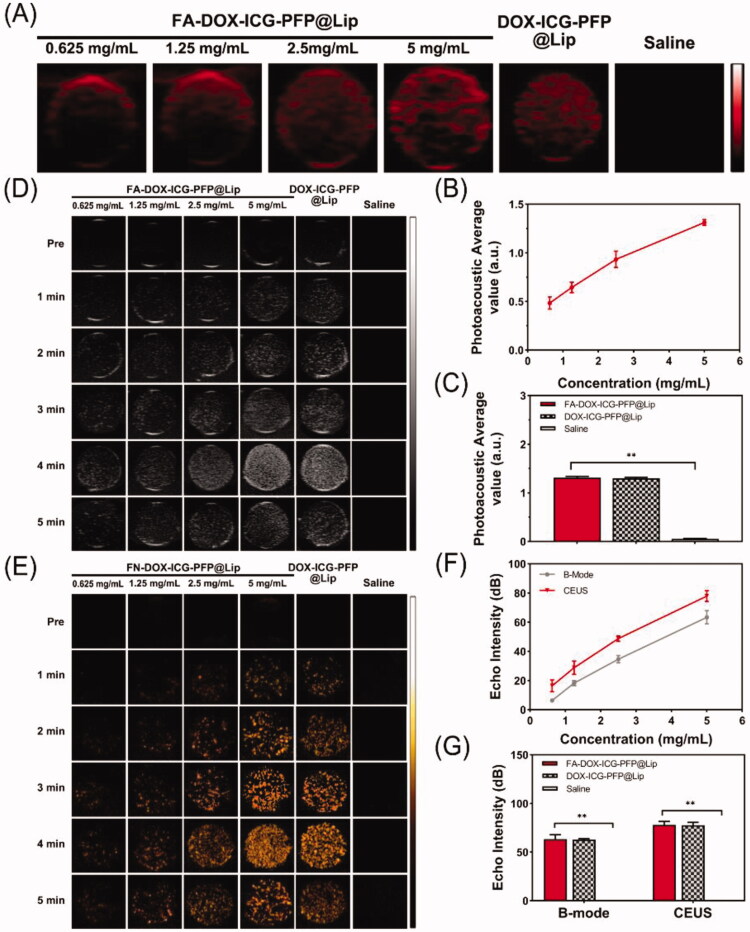
*In vitro* dual-modal imaging. (A) Photoacoustic images of FA-DOX-ICG-PFP@Lip (0.625, 1.25, 2.5, and 5 mg/mL), DOX-ICG-PFP@Lip (5 mg/mL), and saline. (B) Photoacoustic values of FA-DOX-ICG-PFP@Lip (0.625, 1.25, 2.5, and 5 mg/mL). (C) Photoacoustic values of FA-DOX-ICG-PFP@Lip (5 mg/mL), DOX-ICG-PFP@Lip (5 mg/mL), and saline. (D) B-mode and (E) contrast-enhanced ultrasound images of FA-DOX-ICG-PFP@Lip (0.625, 1.25, 2.5, and 5 mg/mL), DOX-ICG-PFP@Lip (5 mg/mL), and saline with laser irradiation at 1 W/cm^2^ for 5 min. (F) Echo intensity of FA-DOX-ICG-PFP@Lip (0.625, 1.25, 2.5, and 5 mg/mL) in B-mode and contrast-enhanced ultrasound. (G) Echo intensities of FA-DOX-ICG-PFP@Lip (5 mg/mL), DOX-ICG-PFP@Lip (5 mg/mL), and saline (**p<.01).

The use of multimodal imaging has received much attention recently (Burke et al., 2017). However, each modality has unique advantages and disadvantages. Dual-modal imaging, such as fluorescence imaging used with MR imaging, computed tomography combined with US, and MR combined with nuclear imaging, have surmounted these problems to some degree (Kim et al., 2011; Lee et al., 2013; Wang et al., 2016; Zhang et al., 2017). PA imaging is relatively new, and its optical imaging characteristics enable high resolution. US is the most widely used imaging method in the clinic. And CEUS, can enhance the detection and visualization of a variety of lesions, including those in liver, breast, and thyroid tissue (Schleder et al., 2015; Xiang et al., 2017; Durot et al., 2018). Therefore, we constructed FA-DOX-ICG-PFP@Lip as contrast agents for dual PA and US imaging. The capabilities of PA imaging were first examined using a PA imaging system. As seen in Figure 3(A,B), the signals were observed to increase in correspondence with increasing concentrations of FA-DOX-ICG-PFP@Lip in the range of 0.625–5 mg/mL. Quantitative analysis (Figure 3(C)) showed that, at 5 mg/mL, FA-DOX-ICG-PFP@Lip and DOX-ICG-PFP@Lip did not differ significantly in PA imaging, while saline alone produced little PA signal. These findings indicated that the FA-DOX-ICG-PFP@Lip was suitable for using as a PA contrast agent.

FA-DOX-ICG-PFP@Lip (0.625-5 mg/mL), DOX-ICG-PFP@Lip (5 mg/mL), and saline were further compared using B-mode and CEUS imaging. As seen in Figure 3(D,E), no US enhancement in either mode was visible before irradiation. Under irradiation for 5 min, a peak of echo intensity was seen at 4 min, after which it declined at 5 min. Therefore, 4 min of irradiation was used for further comparisons. As shown in Figure 3(F), perfect ultrasonic contrast enhancement was obtained in both B-mode and CEUS, with the echo intensity increasing with FA-DOX-ICG-PFP@Lip concentrations over the 0.625–5 mg/mL range. Meanwhile, we observed no significant difference between FA-DOX-ICG-PFP@Lip and DOX-ICG-PFP@Lip in either imaging mode, and saline alone produced little signal. These results suggest that FA-DOX-ICG-PFP@Lip can function as an excellent contrast agent for US due to its phase transition capabilities in response to NIR.

### 3.5. *In vitro* cytotoxicity and cell uptake

**Figure 4.**
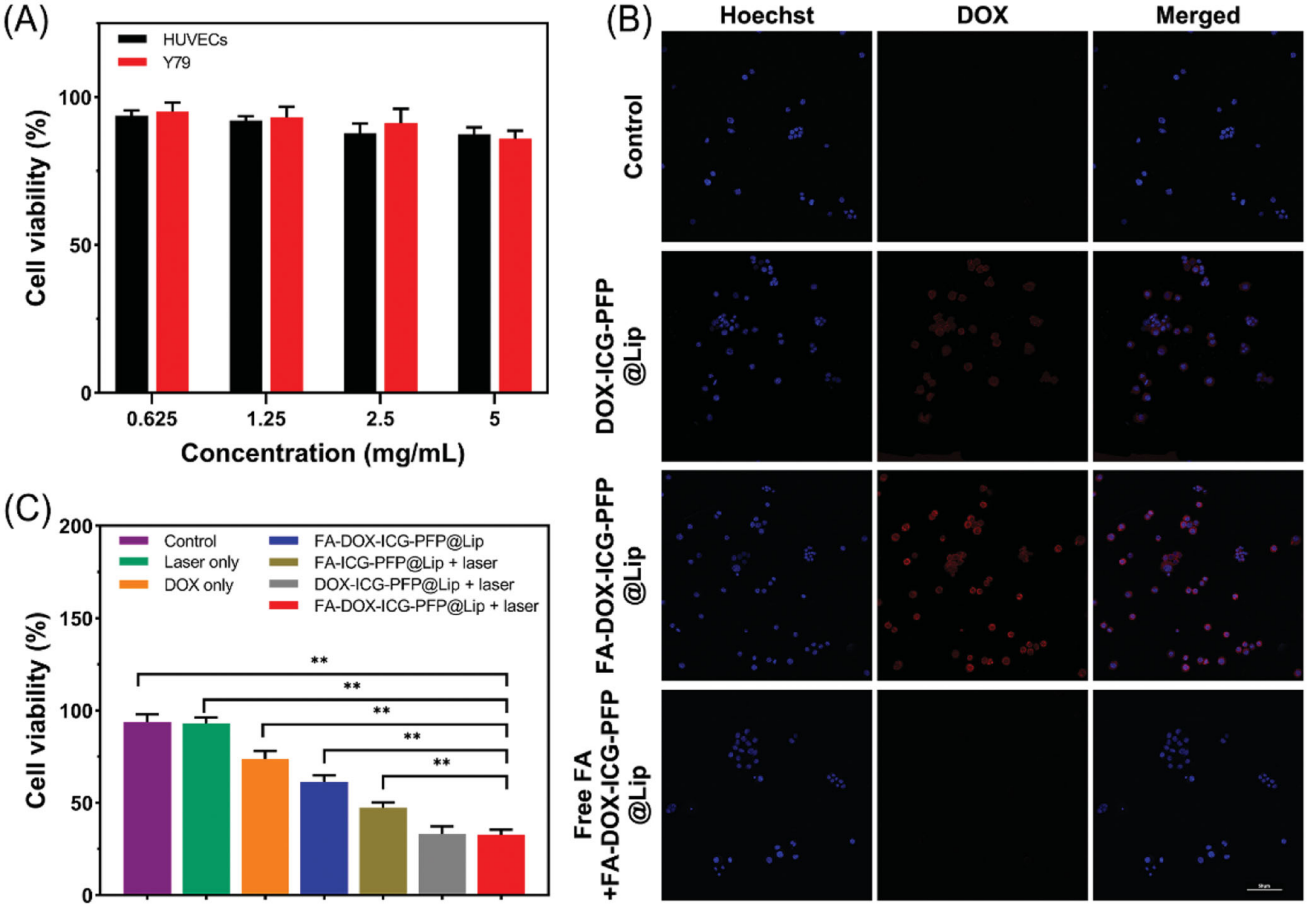
Cytotoxicity, *in vitro* cellular uptake, and anticancer effects of liposomes. (A) Cytotoxicity against HUVEC and Y79 cells after incubation with FA-ICG-PFP@Lip (DOX-free) for 24 hours. (B) Confocal laser scanning micrographs images of Y79 cells after different treatments (nontargeted group: DOX-ICG-PFP@Lip, targeted group: FA-DOX-ICG-PFP@Lip, and antibody antagonist group: free folic acid + FA-DOX-ICG-PFP@Lip). (C) Anticancer efficacy of different therapies against Y79 cells (control, laser only, DOX only, FA-DOX-ICG-PFP@Lip, FA-ICG-PFP@Lip + laser, DOX-ICG-PFP@Lip + laser, FA-DOX-ICG-PFP@Lip + laser) (**p<.01).

The capabilities of FA-ICG-PFP@Lip (DOX-free) as a vehicle for inducing cytotoxic drug-delivery were evaluated in HUVECs and Y79 cells incubated with different concentrations of FA-ICG-PFP@Lip (0.625, 1.25, 2.5, and 5 mg/mL) for 6, 12, and 24 h, using CCK8 for measuring cell viability. As shown in Figure 4(A), there was no visible cytotoxicity, even at the high concentration of 5 mg/mL, indicating the biosafety of FA-ICG-PFP@Lip.

The ability of FA-DOX-ICG-PFP@Lip to target Y79 cells *in vitro* was evaluated by CLSM. Using fluorescence labeling (red from DOX in the liposomes and blue from the Hoechst 33342-labeled nuclei of Y79 cells), we investigated three groups: group 1, DOX-ICG-PFP@Lip incubated with Y79 FR-overexpressing cells; group 2, FA-DOX-ICG-PFP@Lip incubated with Y79 cells; group 3, FA-DOX-ICG-PFP@Lip incubated with Y79 cells with antagonized folate receptors. After three hours, extensive red fluorescence was apparent surrounding the nuclei of the Y79 cells in group 2, with significantly less color seen in group 1. In addition, the red signal was reduced in Y79 cells with antagonized folate receptors (Figure 4(B)). A possible reason for this may be that the FA-containing liposomes bound more effectively to FR-overexpressing Y79 cells. Conversely, as there were fewer FRs available on the cells in group 3, there was a marked reduction in the association of FA-functionalized liposomes. These findings indicate that interactions between FA and its receptor are necessary for adequate liposome phagocytosis and further demonstrate that FA-DOX-ICG-PFP@Lip can target FR-overexpressing cells, thus increasing the amounts of DOX delivered to the cells.

### 3.6. *In vitro* anticancer effect

Based on the demonstrated photothermal properties, effective laser-induced drug release, and ability to target Y79 cells, the antitumor actions of the FA-DOX-ICG-PFP@Lip were investigated in Y79 cells using the CCK-8 assay. The following single or synergistic groups were used: saline (control), saline + laser (laser only), DOX solution (at the liposome-loaded dose, non-targeted chemo), FA-DOX-ICG-PFP@Lip (targeted chemo), FA-ICG-PFP@Lip + laser (PTT), DOX-ICG-PFP@Lip + laser (non-targeted, PTT + chemo), and FA-DOX-ICG-PFP@Lip + laser (targeted, PTT + chemo). As seen in Figure 4(C), the cell viability was similar between the laser group and the control group (*p*>.05), suggesting minimal toxicity under these conditions (808 nm, 1 W/cm^2^, 5 min). Viability in the FA-DOX-ICG-PFP@Lip group was reduced compared with the DOX-only group. It is possible that the FA-functionalized liposomes loaded with DOX may traverse the cell membrane more effectively than free DOX, as the FA-modified nanoparticles enter the cell by receptor-mediated endocytosis (Yang et al., 2012; Zheng et al., 2012). Cell viability was found to be lower in the FA-ICG-PFP@Lip + laser group relative to the FA-DOX-ICG-PFP@Lip group. This suggests that PTT may be necessary for the antitumor effect. This may be due to destruction of expanding microbubbles when the endocytosed liposomes were activated by laser irradiation, leading to microbubble formation and expansion and resulting in cell death. Notably, marked cytotoxicity was seen in the FA-DOX-ICG-PFP@Lip + laser group compared with the FA-DOX-ICG-PFP@Lip and FA-ICG-PFP@Lip + laser groups. The cell viability in the FA-DOX-ICG-PFP@Lip + laser group was 32.67 ± 2.85%, considerably less than in the other groups, apart from the DOX-ICG-PFP@Lip + laser group; this may be because the viability of cells cultured in a tissue culture plate was not affected by ligand–receptor interactions. Overall, FA-DOX-ICG-PFP@Lip together with laser irradiation showed the lowest cell viability and highest rate of apoptosis, indicating effective synergy between ICG, PFP, and DOX, together with laser irradiation.

### 3.7. *In vivo* biocompatibility

**Figure 5.**
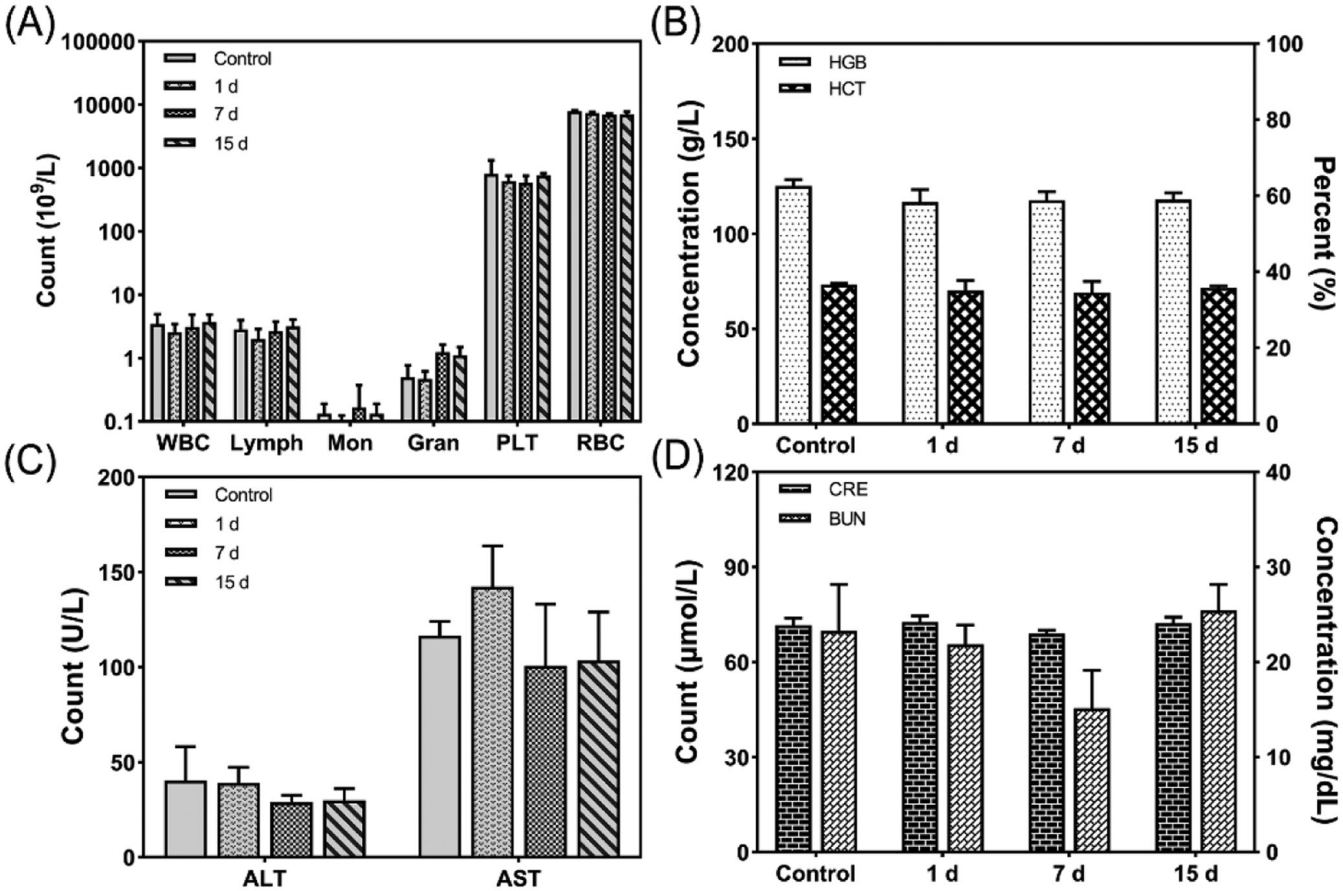
Hematological parameters, liver function, and renal function of the mice after injection of FA-DOX-ICG-PFP@Lip. (A) White blood cell, lymphocyte, monocyte, granulocyte, platelet, and red blood cell counts of the mice 1, 7, and 15 d after liposome injection. (B) Hemoglobin concentrations and hematocrit percentages r 1, 7, and 15 d after liposome injection. (C) Alanine transaminase and aspartate transaminase values 1, 7, and 15 d after liposome injection. (D) Creatinine and blood urea nitrogen values 1, 7, and 15 d after liposome injection. ALT: alanine transaminase; AST: aspartate transaminase; BUN: blood urea nitrogen; HCT: hematocrit; HGB: hemoglobin; PLT: platelet count; RBC: red blood cell count; WBC: white blood cell count.

Although conventional chemotherapy is effective for treating cancer, it has adverse side effects (Iwamoto, 2013). Therefore, the *in vivo* biocompatibility of FA-DOX-ICG-PFP@Lip was investigated in terms of routine blood/serum biochemical parameters. As shown in Figure 5, there was no reduction in either the white blood cell or platelet counts in the FA-DOX-ICG-PFP@Lip group, indicating that there was no significant myelosuppression. In addition, there were no indications that the liposomes affected either liver or kidney function. These results indicate that the drug-delivery system is both safe and biocompatible, and that the drug is transported directly to the tumor without affecting normal tissue or organs (Pérez-Herrero & Fernández-Medarde, 2015).

### 3.8. *In vivo* biodistribution

**Figure 6.**
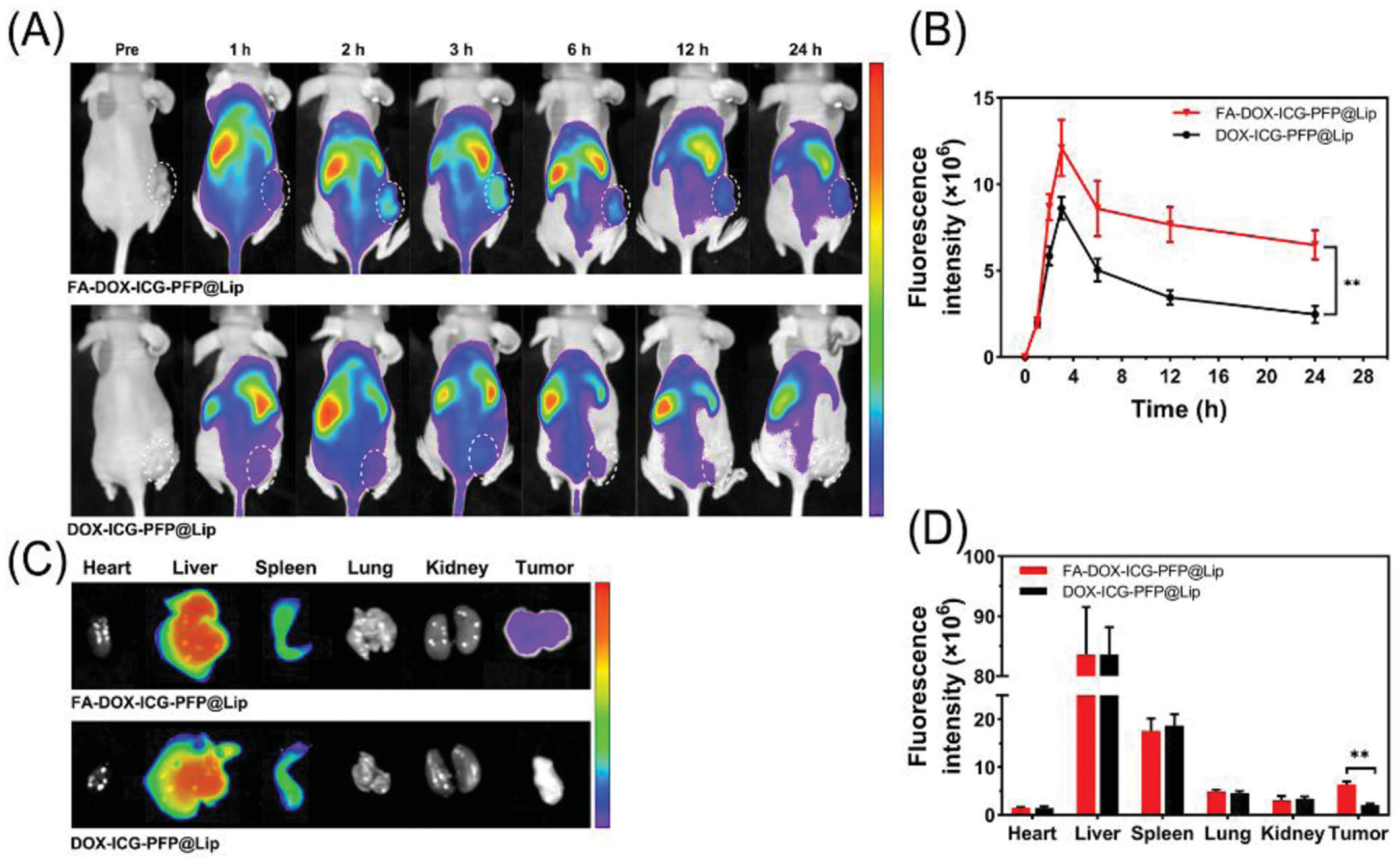
Biodistribution of FA-DOX-ICG-PFP@Lip. (A) Fluorescence images of Y79 tumor-bearing mice at different time points after injection of FA-DOX-ICG-PFP@Lip and DOX-ICG-PFP@Lip. (B) Fluorescence intensity of the tumors at corresponding time points. (C) Biodistribution of DiR-labeled liposomes in major organs and tumors of mice 24 h after injection. (D) Fluorescence intensity in major organs and tumors (**p<.01).

Based on to the demonstrated ability of the FA-DOX-ICG-PFP@Lip to target FR-overexpressing Y79 cells *in vitro*, *in vivo* biodistribution studies were conducted to provide a basis for PA/US imaging and antitumor therapy. FA-DOX-ICG-PFP@Lip and DOX-ICG-PFP@Lip labeled with the NIR probe DiR were injected intravenously into two groups of tumor-bearing mice (2.5 mg/mL, 200 μL, per mouse, respectively). As seen in Figure 6(A,B), there were almost no fluorescent signals visible in either group before administration. Within an hour of injection, increased fluorescence was visible in the tumor areas of both groups, reaching a peak at 3 h and thereafter gradually diminishing although still detectable at 24 h. The fluorescence intensity in the tumor vicinity was higher in the FA-DOX-ICG-PFP@Lip group compared with the DOX-ICG-PFP@Lip group from 1 h to 24 h post-injection. In addition, strong fluorescence was seen in the livers and spleens of both groups, suggesting liposome uptake by the reticuloendothelial system, which is abundant in both organs (Tartaro et al., 2015). The tumors and major organs were then removed for analysis *ex vivo*, showing significantly elevated fluorescence in the tumor tissue of the FA-DOX-ICG-PFP@Lip group than in the DOX-ICG-PFP@Lip group (Figure 6(C,D)). These results demonstrate that despite the efficient accumulation of DOX-ICG-PFP@Lip in tumors through the EPR effect (Björnmalm et al., 2017), FA-functionalized FA-DOX-ICG-PFP@Lip nanoparticles leveraged not only this property but also receptor-ligand interactions for targeted binding, promoting superior accumulation.

### 3.9. *In vivo* PA/US imaging

**Figure 7.**
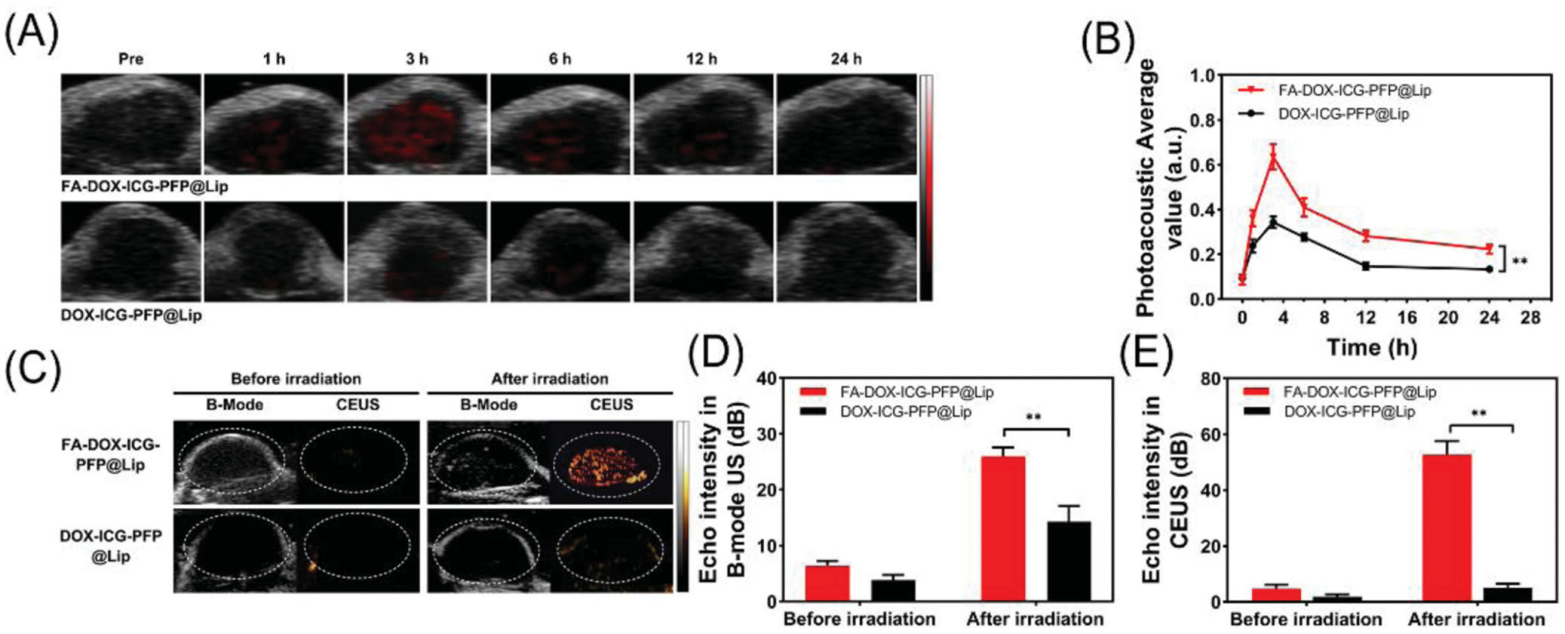
*In vivo* dual-modal imaging. (A) Photoacoustic images of tumors in Y79-tumor-bearing mice after intravenous injection of FA-DOX-ICG-PFP@Lip and DOX-ICG-PFP@Lip at different times. (B) Photoacoustic values of tumors in Y79-tumor-bearing mice at corresponding time points. (C) Ultrasound images of tumors in Y79-tumor-bearing mice after injection of FA-DOX-ICG-PFP@Lip and DOX-ICG-PFP@Lip before and after laser irradiation. (D) Corresponding echo intensities at tumors in Y79-tumor-bearing mice before and after laser irradiation (**p<.01).

Following the demonstrated *in vitro* capabilities of FA-DOX-ICG-PFP@Lip in PA-US imaging, this was further investigated *in vivo*. Two hundred microliters of targeted FA-DOX-ICG-PFP@Lip or nontargeted DOX-ICG-PFP@Lip were injected into the tail veins of tumor-bearing mice, after which they were subjected to PA imaging at different time points. As seen in Figure 7(A,B), there was a weak PA signal, possibly from hemoglobin, in both groups before the injection. Within an hour of the injection, the signal in the tumor region was seen to gradually increase, reaching a maximum at 3 h and then slowly declining. It is worth noting that the signals in the FA-DOX-ICG-PFP@Lip group lasted longer than those in the DOX-ICG-PFP@Lip group. These findings are in agreement with the *in vivo* fluorescence-imaging results and confirmed the effective accumulation of the FA-DOX-ICG-PFP@Lip in tumor tissues. This suggests that FA-DOX-ICG-PFP@Lip may be useful in PA imaging and image-guided tumor therapy.

As FA-DOX-ICG-PFP@Lip is activated by microbubble formation, the utility of liposomes as US-imaging agents was investigated. Three hours after injection of the liposomes, the tumor regions were irradiated (808 nm, 1 W/cm^2^, 5 min). The US images of the tumors in the FA-DOX-ICG-PFP@Lip and DOX-ICG-PFP@Lip groups are shown in Figure 7(C). From Figure 7(D,E), it can be seen that there is clear enhancement in the CEUS mode in the FA-DOX-ICG-PFP@Lip group, in contrast to a poor result in the DOX-ICG-PFP@Lip group. This may be due to both the targeting capabilities of the FA-DOX-ICG-PFP@Lip and more microbubble formation resulting from higher temperatures in the FA-DOX-ICG-PFP@Lip group.

### 3.10. *In vivo* anticancer therapy

**Figure 8.**
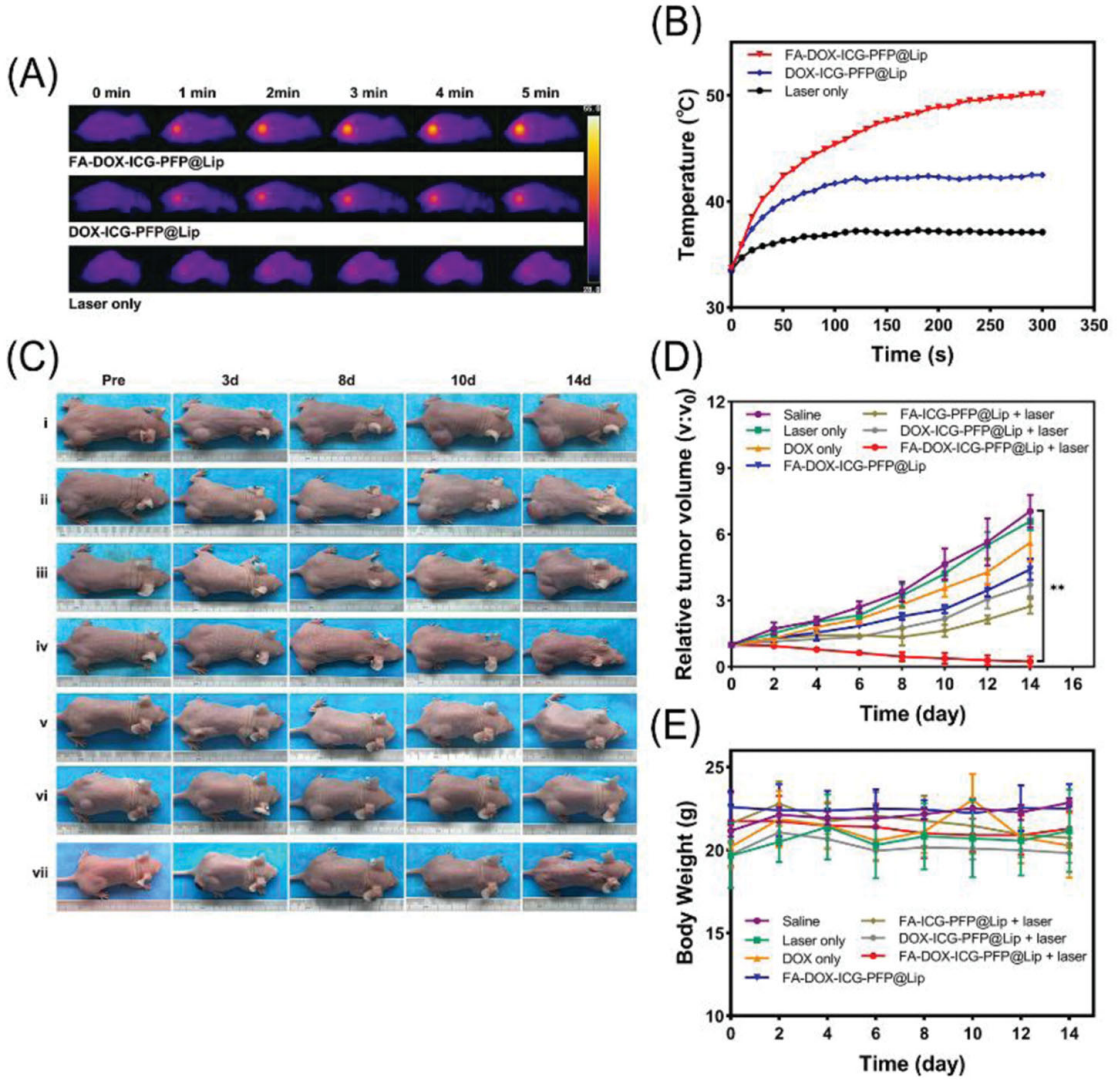
*In vivo* anticancer therapy. (A) Thermal images of tumor-bearing mice 3 h after injection of FA-DOX-ICG-PFP@Lip, DOX-ICG-PFP@Lip, and saline under laser irradiation (1 W/cm^2^, 5 min). (B) Temperature–time curves of the three groups. (C) Photographs of Y79-tumor-bearing mice from seven groups during the 14-d period after different treatments (saline [i], laser only [ii], DOX only [iii], FA-DOX-ICG-PFP@Lip [iv], FA-ICG-PFP@Lip + laser [v], DOX-ICG-PFP@Lip + laser [vi], FA-DOX-ICG-PFP@Lip + laser [vii]). (D) Relative tumor–volume curves of different groups of Y79-tumor-bearing mice. (E) Body weights of mice measured during the 14-d period in different groups (**p<.01).

Considering the good targeting ability and *in vitro* cell killing capability of the FA-DOX-ICG-PFP@Lip, the synergistic anticancer efficacy of FA-DOX-ICG-PFP@Lip was evaluated *in vivo*. First, photothermal conversion *in vivo* was evaluated using a thermal imaging camera. Two hundred microliters of FA-DOX-ICG-PFP@Lip, DOX-ICG-PFP@Lip, or saline were injected into the tail veins of the mice and the tumor regions were irradiated (808 nm, 1 W/cm^2^, 5 min) after 3 h with continuous temperature monitoring. As shown in Figure 8(A,B), the temperature increased significantly in the FA-DOX-ICG-PFP@Lip group, reaching 50.1 °C after 5 min, while the temperature of DOX-ICG-PFP@Lip group only reached 42.5 °C and that of the control group was almost unchanged. This suggests the safety of the laser treatment. Additionally, compared with the FA-DOX-ICG-PFP@Lip group, the temperature was lower in the DOX-ICG-PFP@Lip group after laser irradiation, suggesting greater accumulation of the FA-DOX-ICG-PFP@Lip, leading to higher temperatures.

Next, to further investigate synergistic efficacy, the efficiency of FA-DOX-ICG-PFP@Lip was investigated *in vivo*. Seven groups of tumor-bearing mice were established: saline (control), saline + laser (laser only), DOX solution (at the liposome-loaded dose, non-targeted chemo), FA-DOX-ICG-PFP@Lip (targeted chemo), FA-ICG-PFP@Lip + laser (PTT), DOX-ICG-PFP@Lip + laser (nontarget), and FA-DOX-ICG-PFP@Lip + laser (PTT + chemo). The mice each received 200 μL liposomes or saline. The tumor volumes were subsequently monitored and normalized to relative volumes (*V*/*V*_0_) (Figure 8(C,D)). In the FA-DOX-ICG-PFP@Lip + laser group, there was gradual shrinkage of the tumor with disappearance of the scab. In contrast, in the other groups, the treatment was either ineffective or the tumor recurred. Tumor growth curves showed that the tumor volume in the FA-DOX-ICG-PFP@Lip + laser group decreased gradually, indicative of superior therapeutic effects. In addition, some effect was also seen in the laser-only group, suggesting the involvement of factors other than laser irradiation. Tumor growth in the DOX-only group was slightly reduced, 5.62 ± 0.83 times higher than the initial tumor size, while greater reductions were seen in the FA-DOX-ICG-PFP@Lip group where the tumor was 4.41 ± 0.48 times higher than the initial tumor size, suggested that targeted drug-delivery treatment was more effective than chemotherapy. There were also minimal changes in the body weights of the mice over the course of the treatment, indicating that the FA-DOX-ICG-PFP@Lip and laser were safe and effective (Figure 8(E)).

**Figure 9.**
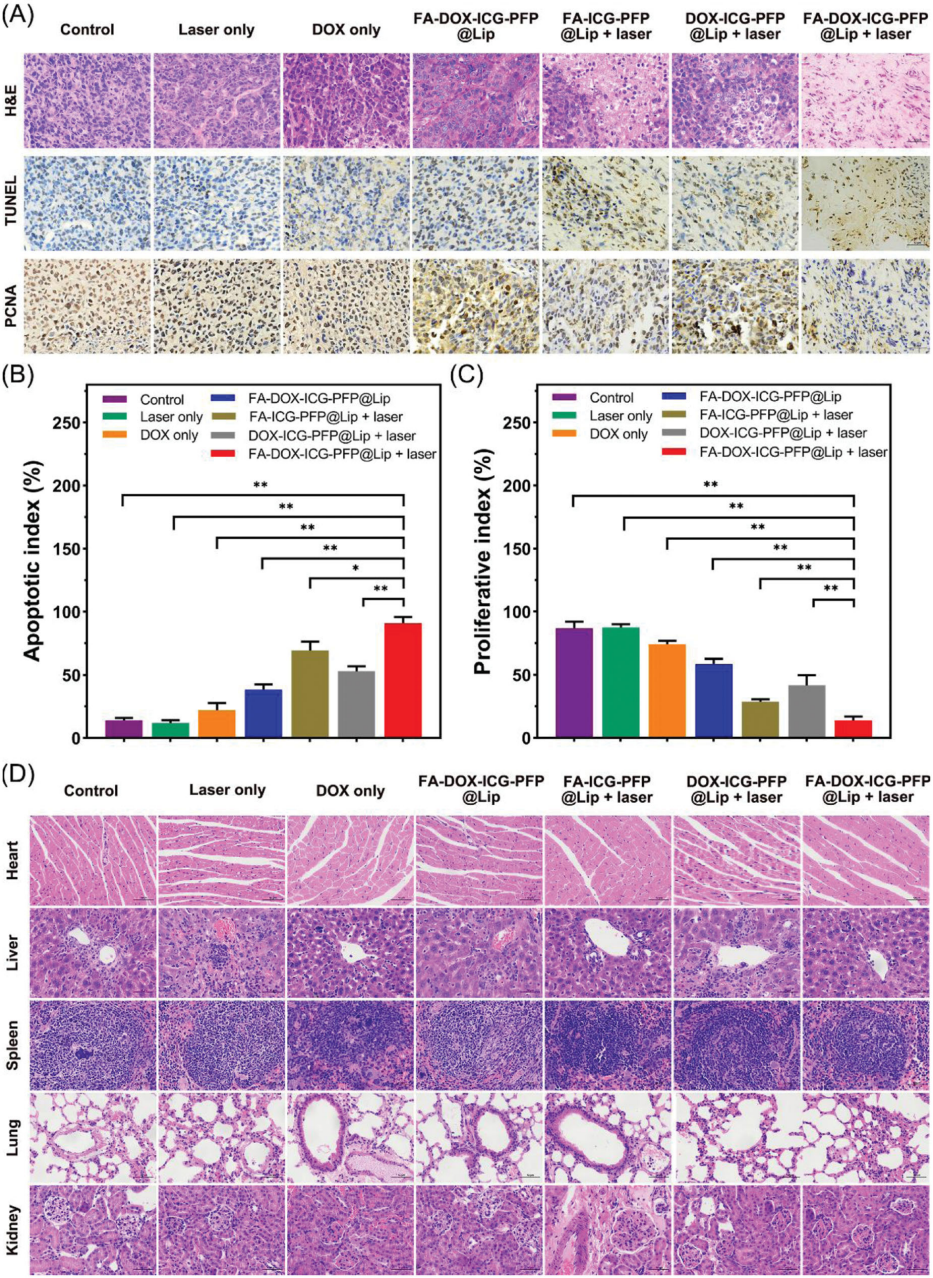
(A) H&E, PCNA, and TUNEL staining in the tumor regions of mice in each group after different treatments. (B) Corresponding TUNEL apoptosis index and (C) PCNA proliferation index in the different groups. (D) H&E staining of major organs (heart, liver, spleen, lung, and kidney) in each group after treatments (*p<.05, **p<.01). H&E: hematoxylin and eosin; PCNA: proliferating cell nuclear antigen; TUNEL: terminal deoxynucleotidyl transferase dUTP nick end labeling.

Staining of tumor sections with H&E, TUNEL, and PCNA further confirmed the synergistic anticancer action of FA-DOX-ICG-PFP@Lip under laser irradiation (Figure 9(A)). Higher levels of apoptosis and necrosis were visible in the FA-DOX-ICG-PFP@Lip + laser group with H&E-staining compared to other groups. The TUNEL assay also showed greater apoptosis and necrosis in other groups, especially in the FA-DOX-ICG-PFP@Lip + laser group (Figure 9(B)), compared to the control and laser-only groups. PCNA assays confirmed these findings, showing that the proliferative index was lowest in the FA-DOX-ICG-PFP@Lip + laser group (Figure 9(C)). These results showed yet again that FA-DOX-ICG-PFP@Lip with laser irradiation was more effective.

### 3.11. Biotoxicity evaluation of chemo/photothermal therapy

The biological safety of the laser- FA-DOX-ICG-PFP@Lip combination was examined by H&E staining of major organs (heart, liver, spleen, lung, and kidney) at treatment completion (Figure 9(D)). There were no visible histological abnormalities seen in any of the groups, indicating the safety and lack of toxicity of the therapy.

## 4. Conclusions

The use of nanoscale drug-delivery systems has been widely investigated in the field of tumor therapy. In this study, we successfully constructed FA-DOX-ICG-PFP@Lip, an FR-targeted laser-activable liposome loaded with DOX and ICG. On accumulation of FA-DOX-ICG-PFP@Lip in tumors via the EPR effect and ligand–receptor interactions, the PFP in liposomes underwent liquid–gas phase transition with 808 nm laser irradiation, thus enhancing US imaging. In addition, ICG loaded in FA-DOX-ICG-PFP@Lip was effective as a contrast agent in PA imaging. Thus, the FA-DOX-ICG-PFP@Lip can be used with PA/US imaging. Moreover, using PA/US guidance, the FA-DOX-ICG-PFP@Lip was shown to be an effective delivery platform for chemotherapeutic drugs and photothermal agents, resulting in superior chemo/PTT for RB when activated by NIR laser irradiation. Furthermore, the nanoplatform was found to be biocompatible both *in vitro* and *in vivo*. These findings indicate the potential of this nanoplatform for cancer treatment.
